# Enhancing near-infrared photoluminescence from single-walled carbon nanotubes by defect-engineering using benzoyl peroxide

**DOI:** 10.1038/s41598-020-76716-9

**Published:** 2020-11-16

**Authors:** Lukasz Przypis, Maciej Krzywiecki, Yoshiaki Niidome, Haruka Aoki, Tomohiro Shiraki, Dawid Janas

**Affiliations:** 1 Department of Organic Chemistry, Bioorganic Chemistry and Biotechnology, Silesian University of Technology, B. Krzywoustego 4, 44-100 Gliwice, Poland; 2 Institute of Physics-CSE, Silesian University of Technology, Konarskiego 22B, 44-100 Gliwice, Poland; 3 Department of Applied Chemistry, Graduate School of Engineering, Kyushu University, 744 Motooka, Nishi-ku, Fukuoka 819-0395 Japan

**Keywords:** Carbon nanotubes and fullerenes, Materials for optics, Synthesis and processing

## Abstract

Single-walled carbon nanotubes (SWCNTs) have been modified with ester groups using typical organic radical chemistry. Consequently, traps for mobile excitons have been created, which enhanced the optical properties of the material. The proposed methodology combines the benefits of mainstream approaches to create luminescent defects in SWCNTs while it simultaneously avoids their limitations. A step change was achieved when the aqueous medium was abandoned. The selection of an appropriate organic solvent enabled much more facile modification of SWCNTs. The presented technique is quick and versatile as it can engage numerous reactants to tune the light emission capabilities of SWCNTs. Importantly, it can also utilize SWCNTs sorted by chirality using conjugated polymers to enhance their light emission capabilities. Such differentiation is conducted in organic solvents, so monochiral SWCNT can be directly functionalized using the demonstrated concept in the same medium without the need to redisperse the material in water.

## Introduction

Since carbon nanotubes (CNTs) were popularized in the early 1990s, many research groups have provided evidence for their extraordinary electrical^1^, thermal^2^, and optical^3^ nature. One of the areas which offers particular opportunities for semiconducting single-walled CNTs (s-SWCNTs) is the field of photonics owing to the presence of bandgap in these materials. As a result, they can fluoresce^4^ over a wide spectral range from visible to the near-infrared^5^ attracting a considerable share of attention to these nanostructures. Unfortunately, studies on this topic revealed that pristine SWCNTs have a low photoluminescence quantum yield (PLQY), estimated at 1.0% in liquid^6^ and about 0.1% in films^7^.

It was recently found that slight functionalization of the material can notably enhance this property^8^. The introduction of sp^3^ defects (evident by the emergence of E_11_^*^ signatures) creates mobile exciton traps of appropriate energy levels, facilitating radiative recombination^9–11^, thereby markedly boosting the s-SWCNT PLQY^10^. To attain such performance, s-SWCNTs are modified by doping with oxygen^12,13^, or they are grafted with aryl or alkyl derivatives. The protocols of SWCNT arylation commonly involve reactions with aryldiazonium salts or^10,14,15^ diazoethers (arenediazoates)^14,16^, whereas alkylation techniques^17^, are generally based on adaptations of Billups-Birch reduction protocols^18^.

Despite the effectiveness of these routines, most of them are limited to water dispersions or water/polar solvent mixtures. Employing aqueous media for such processing is particularly inexpedient in the light of recent milestones achieved on the front of SWCNTs sorting^19^. It was shown that certain conjugated-polymers soluble in organic solvents enable isolation of SWCNTs of particular electrical character or even chirality. Given that polymer-wrapped SWCNTs are compatible with organic solvents, it is highly desirable to develop an effective sp^3^ functionalization method dedicated to SWCNT dispersions in such media. To tackle this problem, Zaumseil and colleagues devised a concept of using diazonium salts outside of the aqueous environment^20^. They utilized crown ether as a phase-transfer reagent to realize the first effective diazonium salts functionalization of SWCNTs in the toluene/acetonitrile mixture. Successful modification of monochiral SWCNTs dispersed with conjugated polymers was confirmed after 16 h of reaction in darkness.

In parallel, grafting of SWCNTs with radicals received a particular share of attention because it can be accomplished at the reduced time, as indicated by Maeda^21^ and Weisman groups^13^. Maeda et al*.* investigated the radical functionalization of SWCNTs under sonication of the SDBS-dispersion of SWCNTs in water^21^. The authors postulated that under these conditions, hydroxyl radicals and hydrogen peroxide are generated, which grafted the SWCNT sidewall to give E_11_^*^ and E_11_^2*^ peaks. Furthermore, Weisman and co-workers demonstrated effective functionalization of SWCNTs using sodium hypochlorite under UV illumination. Oxygen defects were added in less than 1 min^13^. What is more, as recently shown, fluorescent defects can be implanted in SWCNTs by employing photoexcited aromatic compounds^22^. Strong E_11_^*^ and E_11_^2*^ bands emerge rapidly, and their intensity peaks within a few minutes. These strategies show the possibility of efficient, controllable, and rapid functionalization of SWCNTs with radicals without causing collateral damage. We were inspired by these results and decided to explore if the defect chemistry can be done not in the water, wherein it suffers from the reactive nature hydroxyl radical, but in other media^23^.

Herein, we describe our strategy of functionalization of SWCNTs using radical organic reagents in toluene. The aim of this approach was motivated by several reasons. Firstly, we wanted to provide appropriate conditions for controllable defect implantation. For this, we used an organic solvent instead of water, thereby eliminating potential pathways for generating aggressive radicals such as ·OH. Their addition is often hard to control, and hence it results in deterioration of the optical properties of the SWCNTs. Secondly, several techniques of isolation of SWCNTs of particular chirality were recently devised for organic solvents, which employ polymer surfactants^24,25^. It is essential to develop dedicated modification strategies for non-aqueous environments because these polymer-harvested SWCNTs are almost always insoluble in water. Direct grafting of sorted SWCNTs without the need to conduct a tedious surfactant-exchange process to make them water-compatible would be very advantageous. Lastly, by redesigning the functionalization tactic, it may be possible to significantly speed up the procedure compared with other mainstream techniques, most of which require hours^20^ or days^10,15^. To tackle these issues, we introduced luminescent sp^3^ defects directly to polymer-wrapped (6,5) SWCNTs in toluene by exposing them to benzoyl peroxide (BPO). Upon reaching 100 °C, the functionalization is completed in 1 h and results in strong E_11_^*^ (1156 nm) and E_11_^*−^ (1280 nm) light emission signatures. Investigation of the possible type of introduced functional group gave strong evidence that a C–O bond between SWCNT and the substituent is formed for the first time, creating the previously unreported type of trap for mobile excitons.

## Results and discussion

We started the study by analyzing the composition of the starting material: (6,5)-enriched CoMoCAT SWCNTs (Fig. 2). Water and toluene dispersions facilitated by sodium cholate (SC) as well as poly(9,9-dioctylfluorenyl-2,7-diyl) and bipyridine copolymer (PFO-Bpy), respectively, were analyzed.

**Figure 1:**
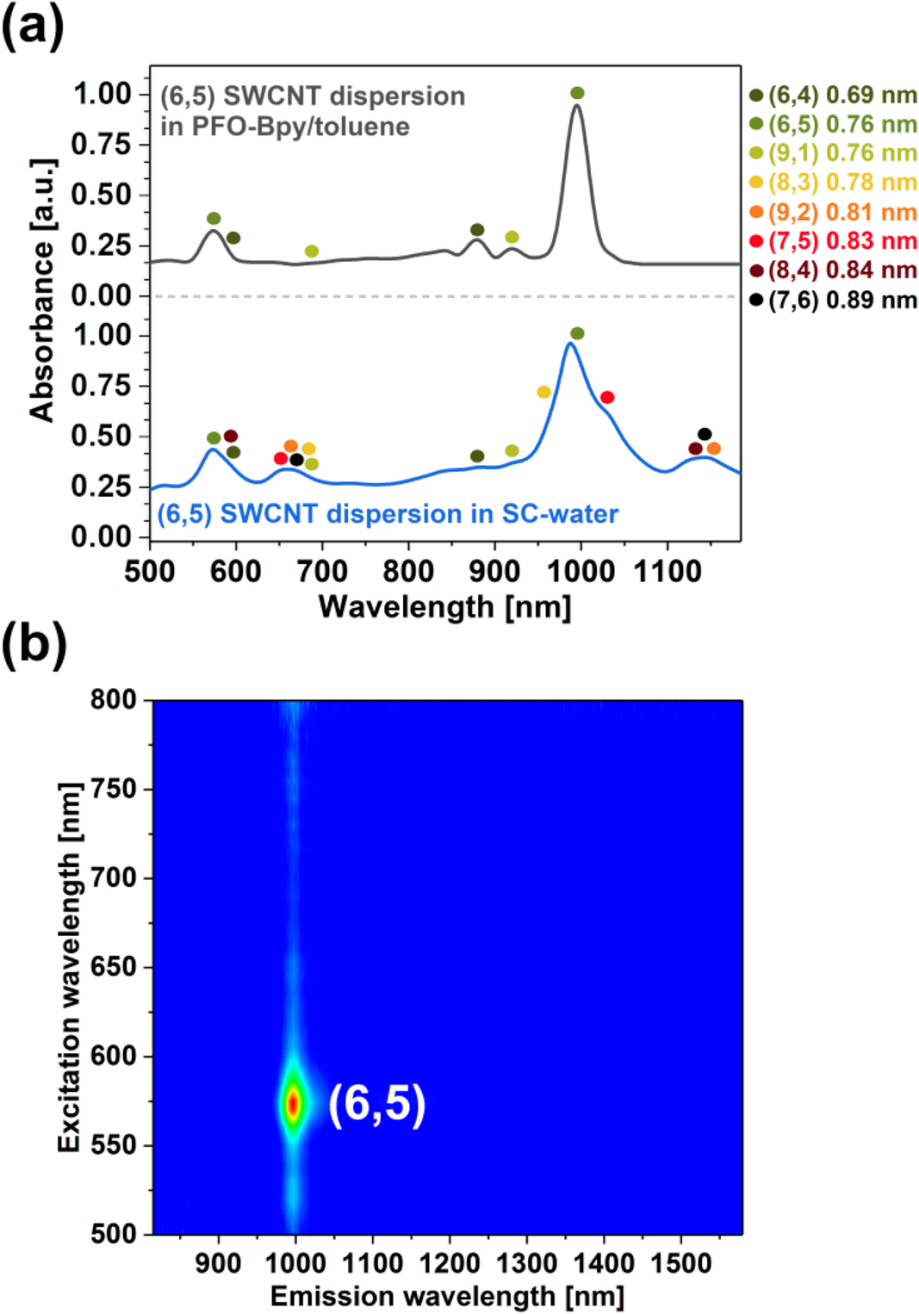
Characterization of the parent material. (**a**) Absorption spectra of (6,5)-enriched SWCNTs dispersed in 2% sodium cholate aqueous solution or PFO-Bpy/toluene solution. (**b**) 2D PLE map of the (6,5)-enriched SWCNT dispersion prepared by PFO-Bpy in toluene.

The parent material in water was predominantly made of (6,5) species, as expected, but other chiralities were also detected. The enclosed absorption spectrum of SWCNT aqueous dispersion of SC illustrated that (6,4), (7,5), (7,6), (8,3), (8,4), (9,1), and (9,2) species were also present (Fig. 1a, blue curve), which was supported by the corresponding 2D PLE mapping (Fig. S1).

Recently, it was reported that upon the combination of raw SWCNTs with a copolymer of poly(9,9-dioctylfluorenyl-2,7-diyl) and bipyridine (PFO-Bpy), (PFO-Bpy, instead of SC) (6,5) SWCNTs can be preferentially extracted to toluene^20,26^. We decided to employ this approach because, in addition to chiral-selectivity of PFO-Bpy in toluene, it was an appropriate medium to solubilize organic radical precursors at concentrations on the order of a few grams per liter^24^. The extraction of (6,5) SWCNTs was successful judging by the presence of sharp E_11_ and E_22_ optical transitions in the absorption spectrum (Fig. 1a, grey curve) as well as the absence of signatures from other SWCNT chiralities in the 2D PLE map (Fig. 1b) at the characteristic wavelengths. Only trace amounts of (6,4) and (9,1) SWCNTs were detected in the absorption spectra, as reported previously for a similar separation routine using PFO-Bpy^20^. We deduced that the selected conjugated polymer exhibits selectivity for SWCNTs of particular diameter distribution because these three SWCNT types had a similar size. Furthermore, the E_11_ peak of (6,5) SWCNTs was found at a relatively long wavelength (997 nm), which followed previous findings for toluene-based dispersions of CNTs of (6,5) type^20^. For water-based dispersions, wherein typical surfactants are employed, the E_11_ peak of these species is usually located between 980^27^ and 990 nm^13^. Therefore, besides solvent effects, the observed redshift proved that PFO-Bpy copolymer was bound tightly to the surface of the highlighted SWCNT types^28,29^, explaining its high differentiation selectivity.

**Figure 2:**
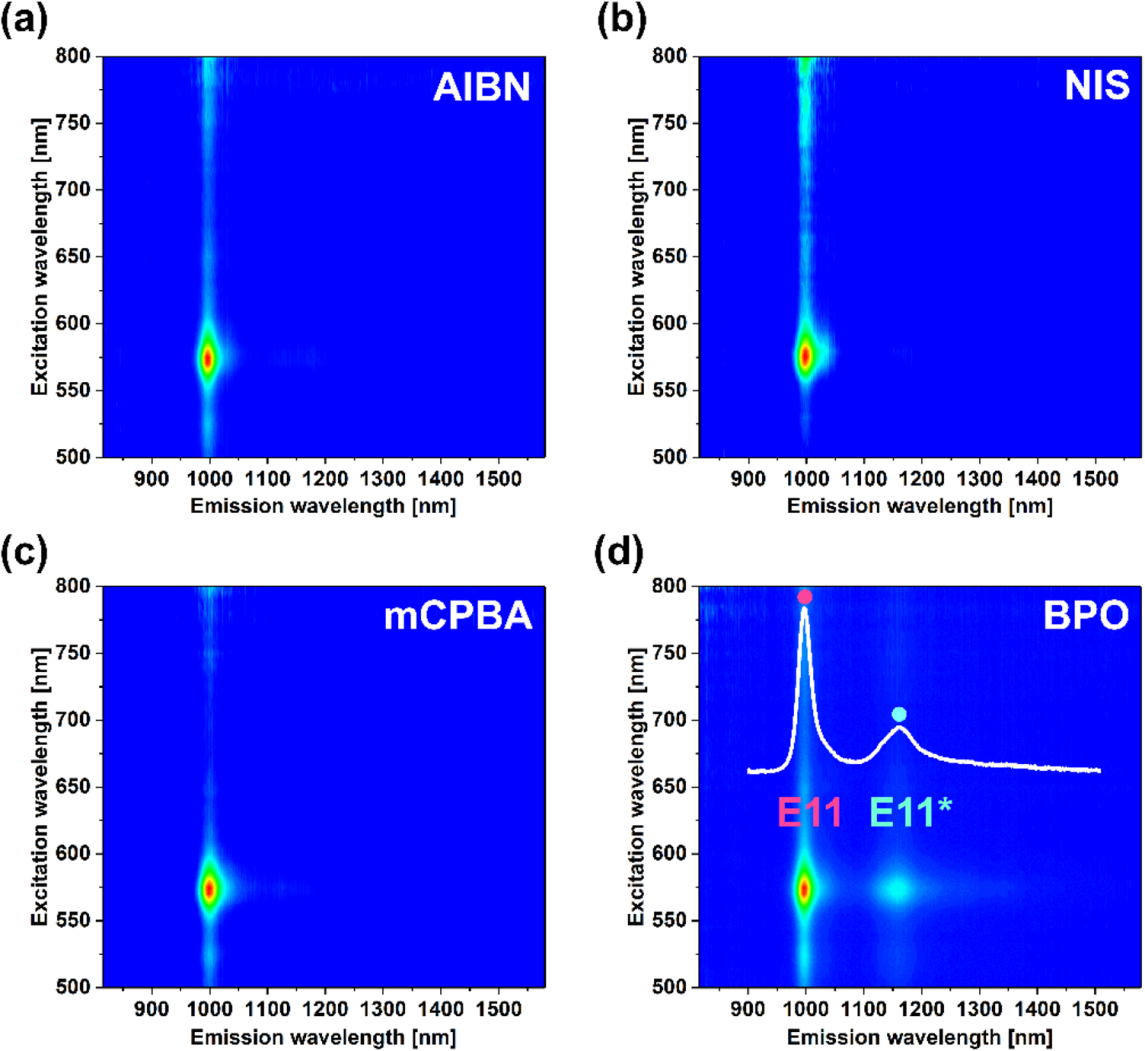
2D PLE maps after treatment of the (6,5)-enriched SWCNT toluene dispersion with various radicals (**a**) AIBN, (**b**) NIS, (**c**) mCPBA, and (**d**) BPO. The inset shows a single PL spectrum collected at 575 nm excitation wavelength extracted from the corresponding map.

Our method for creating luminescent sp^3^ defects in polymer-wrapped SWNTs relied on the addition of preformed organic radical reagents to (6,5) SWCNTs in toluene. First, we evaluated four typical sources of radicals employed in organic chemistry: azobisisobutyronitrile (AIBN), *N*-iodosuccinimide (NIS), *meta*-chloroperoxybenzoic acid (*m*CPBA), and benzoyl peroxide (BPO). 2D PLE maps of the dispersions after the reactions carried out for 1 h at 100 °C are given in Fig. 2. The results provided evidence that the functionalization was successful only in the case of BPO. For this compound, a faint E_11_^*^ defect peak was detected at 1156 nm in addition to the inherent E_11_ signature. Functionalization attempts using other radical reagents did not produce the desired modification.

The organic radicals which we tested can be divided into two types. According to the way they are initiated, they work either by homolytic or heterolytic bond cleavage. AIBN and BPO start the radical reaction by the homolytic breaking of the bond, whereas *m*CPBA and NIS by heterolytic. In the case of homolytic dissociation, it is possible to characterize the reaction by a specific parameter s*.* decomposition rate *k*_*d*_ [s^−1^].

At the temperature of our experiment (100 °C), the values of these parameters for AIBN and BPO are 1.5 × 10^−3^ and 5.0 × 10^−4^ s^−1^, respectively, as reported for benzene^30^. In light of the foregoing, we believe that the desired functionalization occurs when the radical forms at the optimum rate. Too slow pace does not promote the generation of the reactive species at considerable speed, whereas too fast causes self-termination of created radicals. We hypothesize that is the reason why the application of AIBN was unsuccessful. Comparing the decomposition rates shows that AIBN generates radicals three times faster than BPO under the same conditions, so they cannot be used effectively for grafting.

Furthermore, NIS and *m*CPBA have a different radical nature. Firstly, NIS is an ambiguous reagent, and depending on the reaction conditions, it can play the role of an electrophile or radical reservoir. For NIS to induce a radical pathway, a trigger such as light or sufficiently high temperature^31,32^ is required. It is not uncommon to employ UV illumination or temperatures above 150 °C to initiate the radical cascade. That may justify why it was not successful for the functionalization of SWCNTs under the thermal conditions of this study (only 100 °C). The stimulus was not strong enough to release the organic radicals from NIS. Secondly, *m*CPBA is a radical reagent that is typically induced by acid–base treatment. In this case, appropriate conditions for the radical generation were not established in our system too, which explains the observed lack of reactivity of *m*CPBA with SWCNTs. Since just BPO was successful in the explored parameter space, it was employed for further experiments.

An essential practical note is that toluene not only facilitated the dissolution of polymer-wrapped SWCNTs, but it also offered a proper reaction route when BPO was utilized. The functionalization was excessive when we tried an analogous functionalization attempt using SWCNT aqueous dispersion and BPO. In this case, the material lost light-emission capabilities altogether (Fig. S2). The action of BPO in water resulted in the generation of an abundance of aggressive hydroxyl radicals, which readily attached to the SWCNT side wall and disrupted the optical properties of the material. Conversely, when toluene was selected as a medium, it enabled the controllable addition of the organic group onto the SWCNT surface due to the favorable decomposition pathway of BPO.

**Figure 3:**
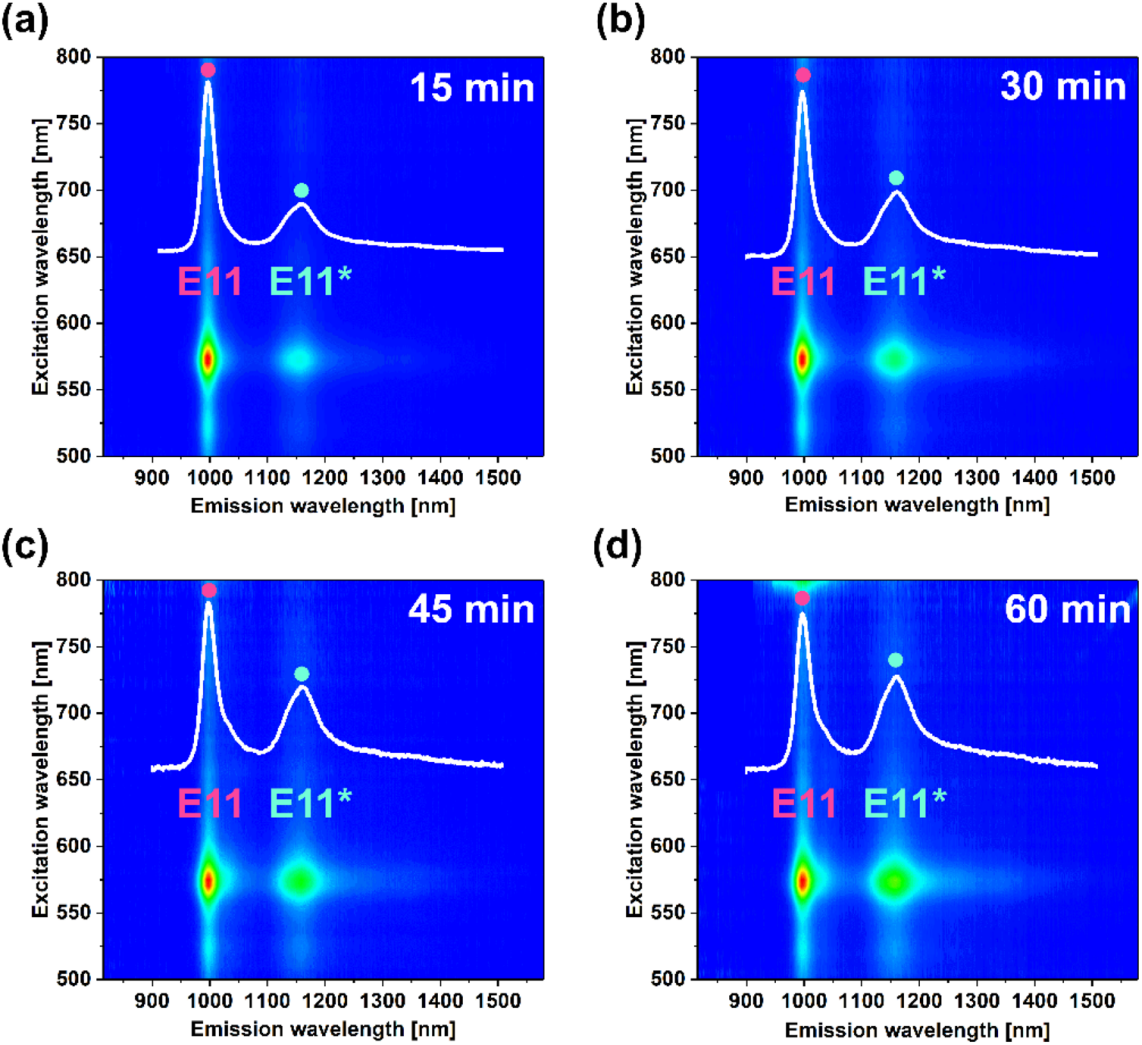
2D PLE maps of the (6,5)-enriched SWCNT dispersion treated with BPO in toluene at 100 µg/mL at 100 °C for various times (**a**) 15 min, (**b**) 30 min, (**c**) 45 min, and (**d**) 60 min. The inset shows single PL spectra collected at 575 nm excitation wavelength extracted from the corresponding maps.

We then studied the impact of the reaction time on the ability of SWCNT to luminescence (Fig. 3). As the time of the reaction was increased from 15 to 60 min, the E_11_^*^ defect peak became brighter. What is more, its position shifted from 1156 to 1160 nm. Two conclusions could be made at this point. Firstly, the selected concentration of 100 µg/mL for BPO was insufficient to reach optimum brightness at the specified time. No signs of photoluminescence quenching could be detected after 60 min; therefore, either longer time or higher concentration of the radical compound should be employed. Secondly, the origins of the observed E_11_^*^ shift could not be ascribed with certainty since the shape of the spectrum was only modified to a small extent under these conditions.

**Figure 4:**
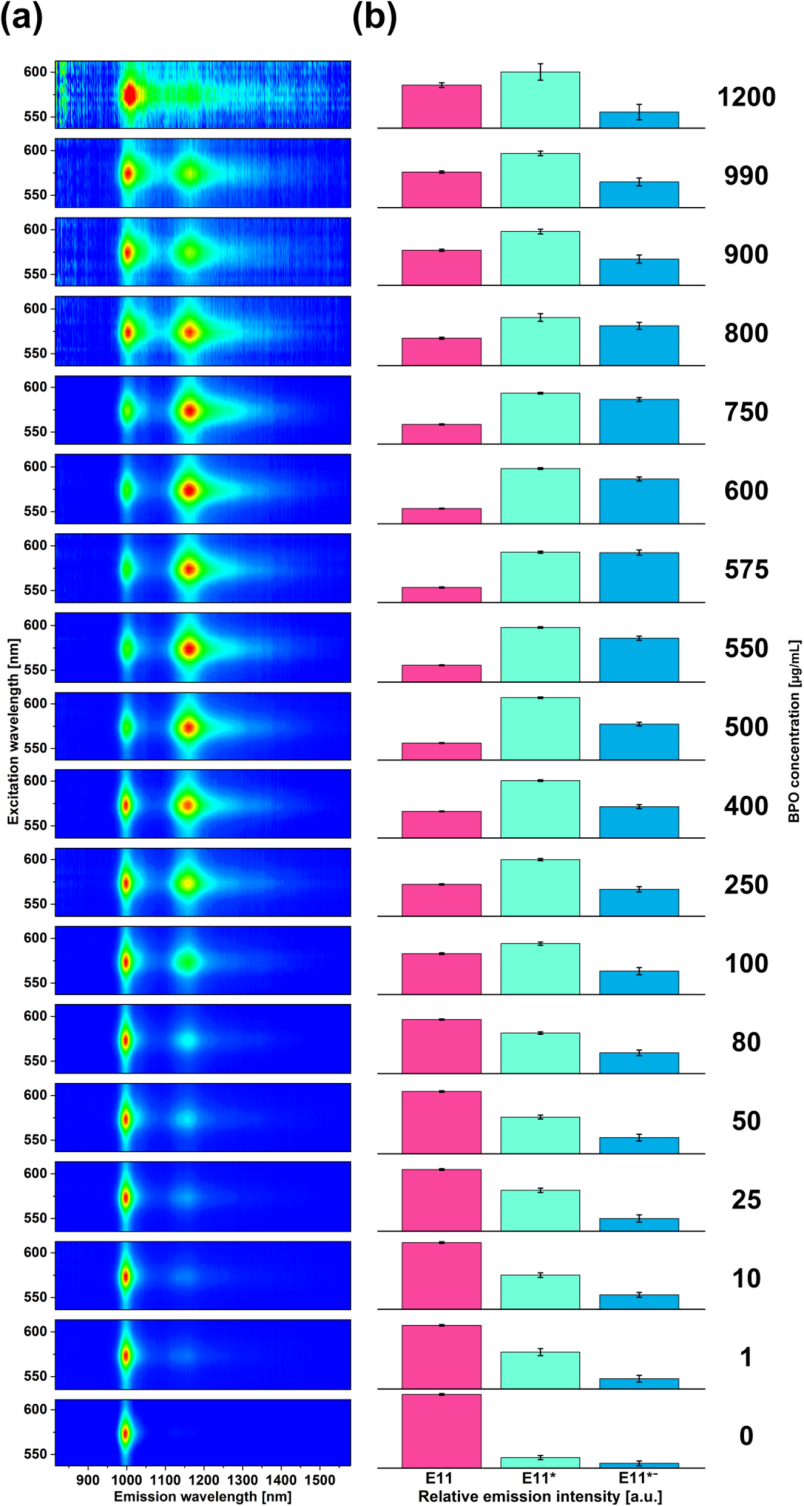
(**a**) 2D PLE maps of the (6,5)-enriched SWCNT dispersion treated with various BPO concentrations in toluene at 100 °C for 1 h. (**b**) deconvolution of the relative intensities of the constituting E_11_, E_11_^*^, and E_11_^*−^ peaks.

To study the phenomenon in greater detail, we decided to conduct functionalization over a wide BPO concentration range from 1 to 1200 µg/mL (Fig. 4a). We observed a clear dependence of the concentration of radicals on the optical properties of the material. Up to 600 µg/mL of BPO, the intensity of the defect peak E_11_^*^ increased, and then it started to decrease. Exceeding this concentration threshold made the material over-functionalized, and hence the PL was quenched. Surplus of defects in the SWCNTs promoted non-radiative recombination of excitons, thereby hampering the light emission capabilities of the material.

**Figure 5:**
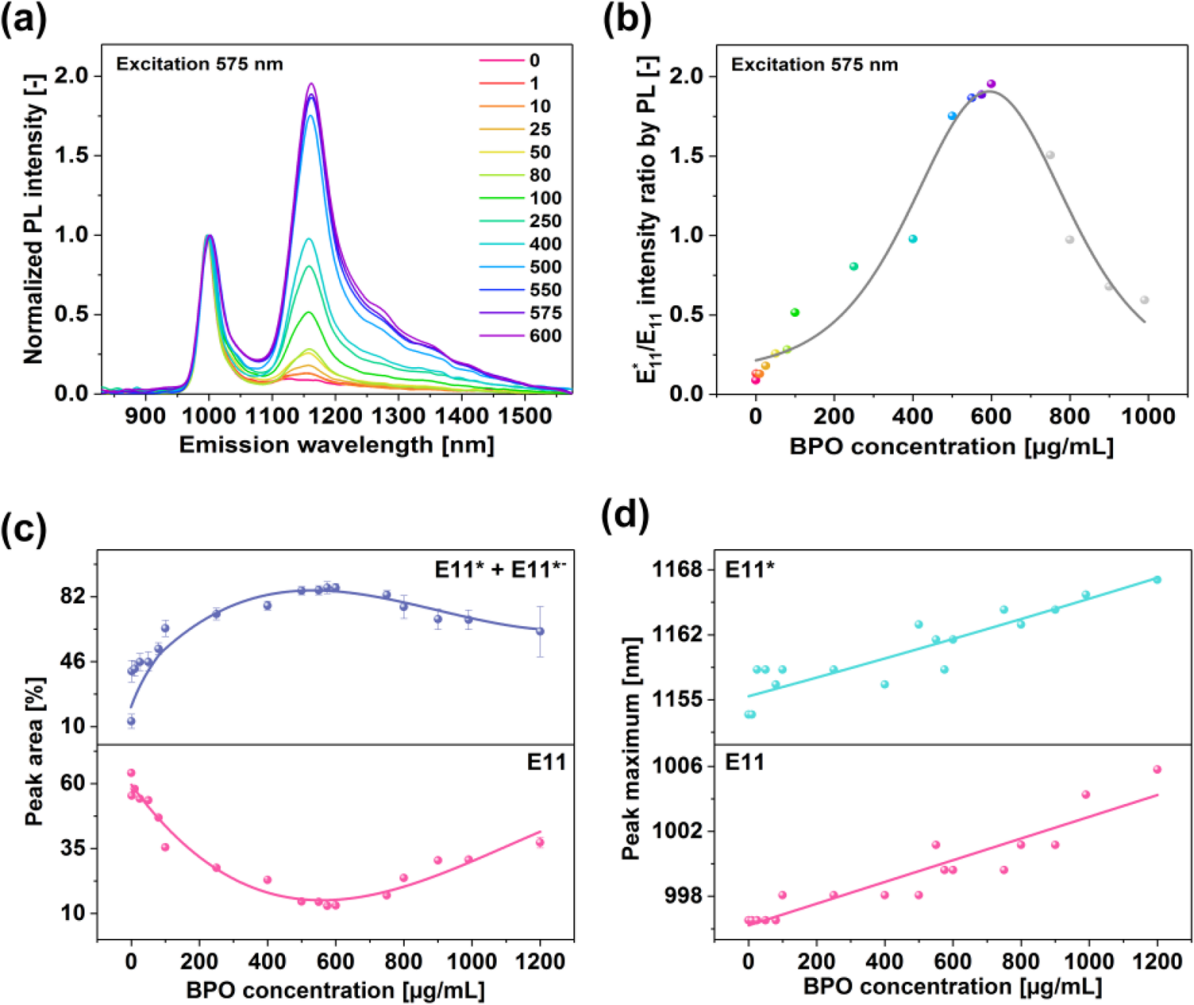
(**a**) Single PL spectra of SWCNTs collected at 575 nm excitation as a function of employed BPO concentration, (**b**) calculated ratios of E_11_^*^/E_11_ intensities as a function of employed BPO concentration, (**c**) the intensity of E_11_ and E_11_^*^ + E_11_^*−^ peaks as a function of employed BPO concentration, and (**d**) position of maxima of E_11_ and E_11_ peaks as a function of employed BPO concentration.

Closer investigation of the acquired 2D PLE maps showed a notable presence of the deep trap states E_11_^*−^ resulting from different arrangements of the grafted functional groups on the surface of the SWCNT^5,33,34^. The tail of the E_11_^*−^ signature extended up to about 1550 nm (Fig. 5a). Because of that, despite the low maximum intensity of this peak (barely distinguishable in 2D PLE maps), its relative share in the PL properties was quite large. Under the optimum conditions of 600 µg/mL of BPO, the integrated intensity of E_11_^*−^ was almost equal to that of E_11_^*^ (Fig. 4b). This finding suggests that this reaction system gave many possible arrangements of functional groups on the SWCNT sidewall. Please refer to Fig. S3 to see how the areas of individual peaks E_11_, E_11_^*^, and E_11_^*−^ were resolved.

Nevertheless, since the absorption spectrum stayed intact after functionalization, we concluded that the functionalization degree after the treatment was low (Fig. S4). Moreover, the modification was accompanied by the increase in the D/G^+^ ratio from 0.043 to 0.131 (Fig. S4b,c), which stayed within the expected range of introduced disorder established by other reports^8^. Regarding the E_11_^*^/E_11_ ratio, the E_11_^*^ defect-peak was up to twice as strong as the inherent E_11_ signature upon modifying the surface. Such an effect was attained even in the absence of electron-withdrawing functional groups on the aryl ring (Fig. 5b). Had we introduced them to the radical precursor, the ratio would probably be higher, as reported for a similar SWCNT grafting approach^20^. The twofold enhancement was nonetheless appreciable. Furthermore, once the concentration of BPO was exceeded beyond 600 µg/mL, the E_11_^*^/E_11_ ratio rapidly declined. The same behavior was noted when comparing the combined E_11_^*^ + E_11_^*−^ intensities against that of E_11_ (Fig. 5c).

Lastly, the position of E_11_ and E_11_^*^ peaks was linearly dependent on the degree of functionalization (Fig. 5d). The latter peak, however, was more sensitive than the former one. The E_11_^*^ defect-peak was redshifted by 12 nm (from 1156 to 1168 nm), whereas the inherent E_11_ signature moved by just 5 nm (997–1004 nm) as the concentration of employed BPO was increased. In other studies, the position of these peaks was invariant of reactant concentration^35^, or these peaks moved in the opposite direction (away^36^ and towards^20^ each other). Our results contrasted with the previously reported work, so we suspected a new type of SWCNT functionalization.

We first noted that the new PL peak arose from the modification of SWCNTs, not from BPO in PFO-Bpy/toluene solution as their combination did not give any peaks in the PLE map in the absence of SWCNTs (Fig. S5). The lack of E_11_^*^ and E_11_^*−^ signals in that PLE map confirmed that recorded signatures came indisputably from the interaction of BPO with SWCNTs. Secondly, the position of the defect peak was strongly red-shifted from that of *O*-doped (6,5)-SWCNTs (1156 nm vs. 1120 nm^37^), which suggested that oxidation of SWCNTs by BPO was an unlikely cause for the observed effect.

**Figure 6:**
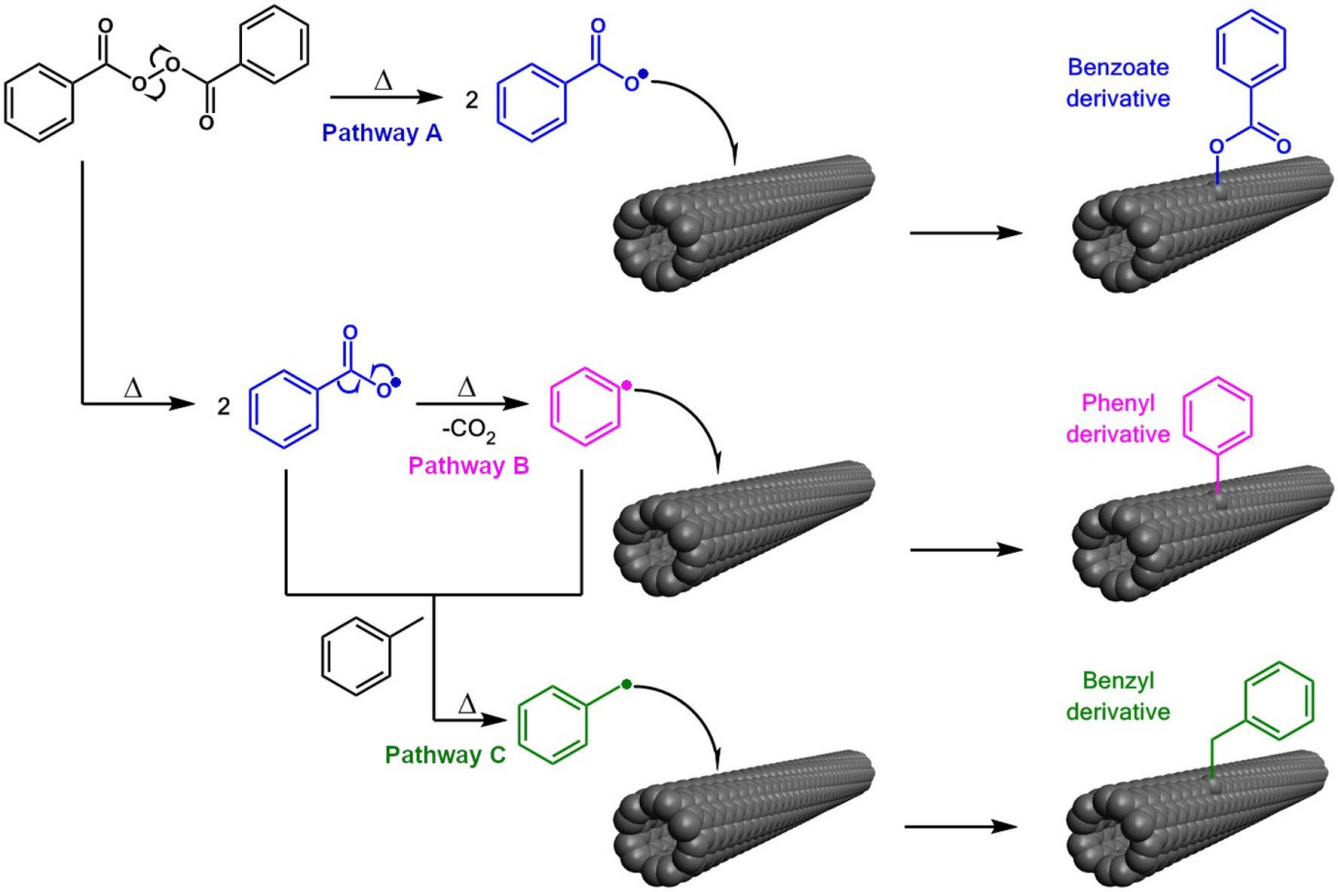
Proposed possible mechanisms of SWCNT functionalization by BPO in toluene, leading to ester, aryl, and arylalkyl derivatives, respectively. PFO-Bpy is not shown on the surface of SWCNTs for the sake of clarity.

To understand this phenomenon, we considered three independent reaction pathways (Fig. 6). In every case, in the first step, it was assumed that BPO thermally dissociated to benzoyloxy radicals. Then, the benzoyloxy radicals could react with SWCNTs directly to create previously unreported ester modification (Pathway A). In the second possible mechanism, benzoyloxy radicals could rearrange to phenyl radicals upon the expulsion of CO_2_ to give phenyl-modified SWCNTs (Pathway B). The position of the defect PL peak in this case defects should be in the same spectral range ca. 1160 nm as in recent a contribution demonstrating grafting of SWCNTs by diazonium salts in toluene^20^. Since we detected the E_11_^*^ peak maximum in this range, this reaction route could not be dismissed. The last proposed possibility (Pathway C) involved the formation of benzyl radicals from the reaction of phenyl or benzoyloxy radical with toluene. In such a case, the benzyl derivative of SWCNTs would be formed. Unfortunately, the position of the defect peak of such chemical arrangement in toluene is not available in the literature as well, so we could not eliminate this possibility either. However, Maeda and co-workers showed that SWCNTs could be grafted with benzyl groups by reductive alkylation. Dispersions of modified SWCNTs prepared this way gave E_11_^*^ signature at about 1100 nm, which corresponded to a Stokes shift of ca. 125 nm^38^. In our case, the Stokes shift was about 160 nm, so even considering solvent and polymer-wrapping effects, the last proposed pathway was less likely.

**Figure 7:**
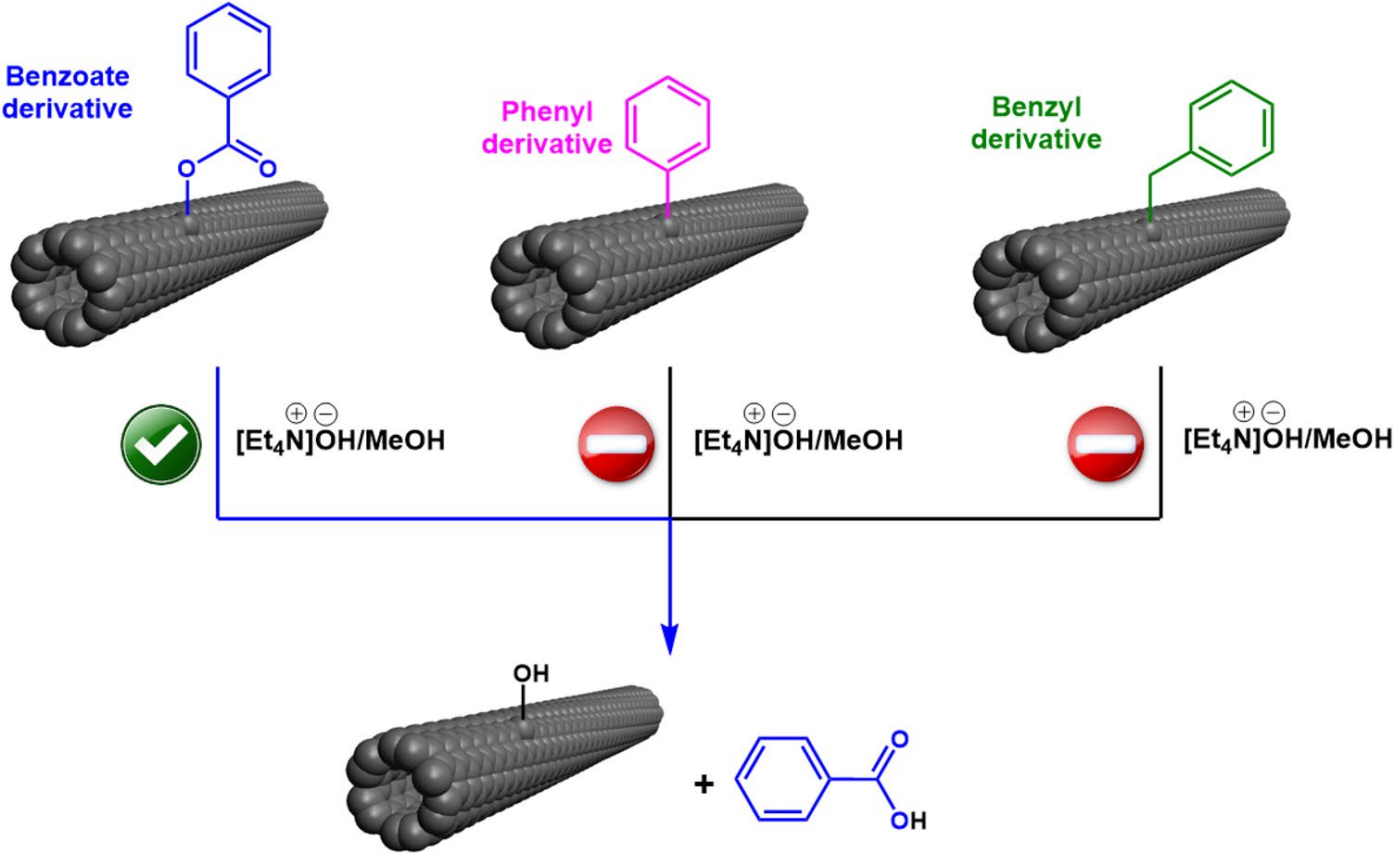
Basic hydrolysis of possible functional groups grafted onto the SWCNT surface by BPO in toluene. PFO-Bpy is not shown on the surface of SWCNTs for the sake of clarity.

**Figure 8:**
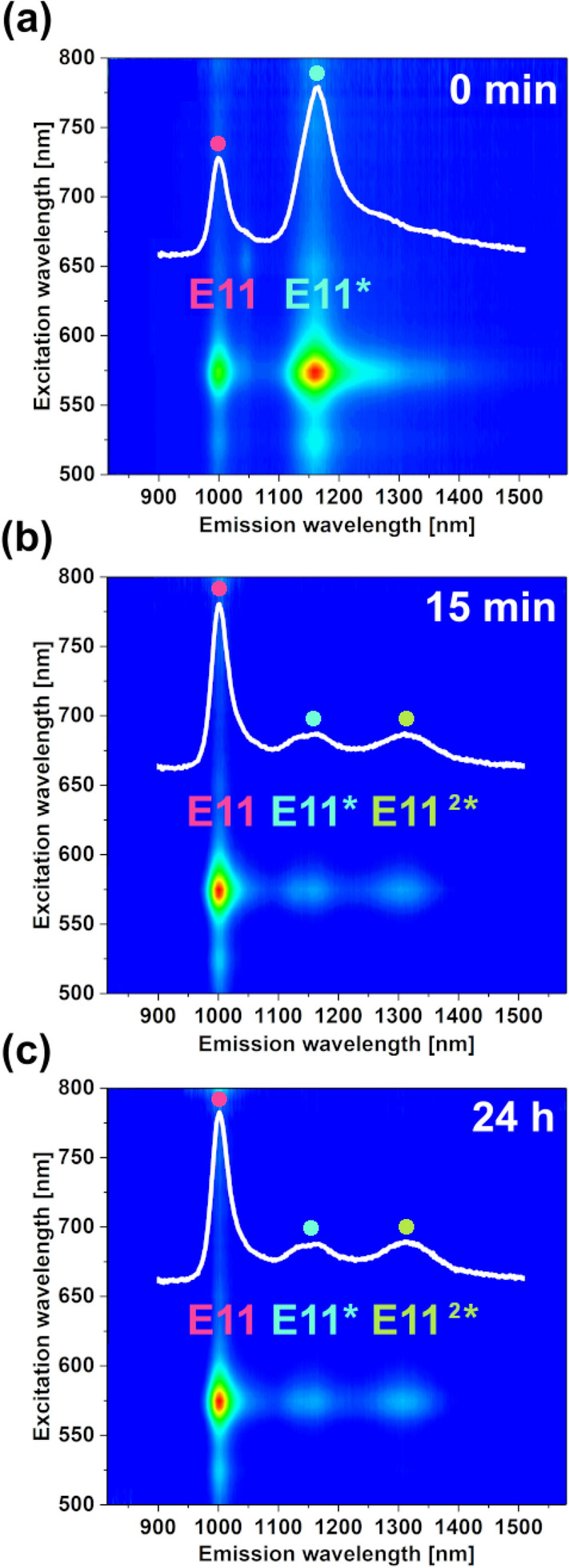
2D PLE maps of (**a**) SWCNT dispersion after functionalization with BPO in toluene at 500 µg/mL concentration for 1 h. Treatment of this sample with [Et_4_N]OH in methanol for (**b**) 15 min and (**c**) 24 h. The insets show corresponding single PL spectra of SWCNTs collected at 575 nm excitation.

To work out the functionalization mechanism and the type of functional group anchored on the SWCNT surface, we had to conduct additional experiments. If the first synthetic route was correct (Pathway A), we could expect that the ester bond between the aryl group and SWCNT could be dissociated by hydrolysis to give corresponding alcohol and carboxylic acid under basic conditions (Figs. 7, 8). This process, in turn, should change the spectral characteristics of the E_11_^*^ signal. Such a reaction could not occur for the other two possible functionalization types (Pathways B and C) because the aryl groups in both these cases were attached with non-labile C–C bonds.

It was challenging to select an appropriate base for toluene because most of them were sparingly soluble in this medium. Therefore, we decided to use ammonium hydroxide in methanol as a co-solvent for SWCNT toluene dispersion. We witnessed rapid spectral response after the treatment of the modified SWCNTs (Fig. 8a) with the base solution at room temperature after just 15 min (Fig. 8b). The suppression of the E_11_^*^ signal was observed at 1156 nm, and additionally, a new signal at 1306 nm of comparable intensity was recorded. The created signature resembled the E_11_^2*^ peak caused by the proximal modification of the SWCNTs^15^. Moreover, the E_11_ signature was restored.

The results gave us more certainty that ester bonds indeed connected substituents to the SWCNT sidewall. Once these bonds were hydrolyzed, the aryl groups were released from the SWCNTs. Simultaneously, hydroxyl moieties took their positions on the SWCNT surface following the well-established mechanism of ester hydrolysis. This reasoning could explain the observed changes to the optical properties of the material gauged by 2D PLE. It has to be noted that such an outcome simultaneously eliminated the possibility of the functionalization to proceed via the phenyl and benzyl radical routes (Pathways B and C) because a simple base treatment could not detach these groups from the SWCNT surface.

**Figure 9:**
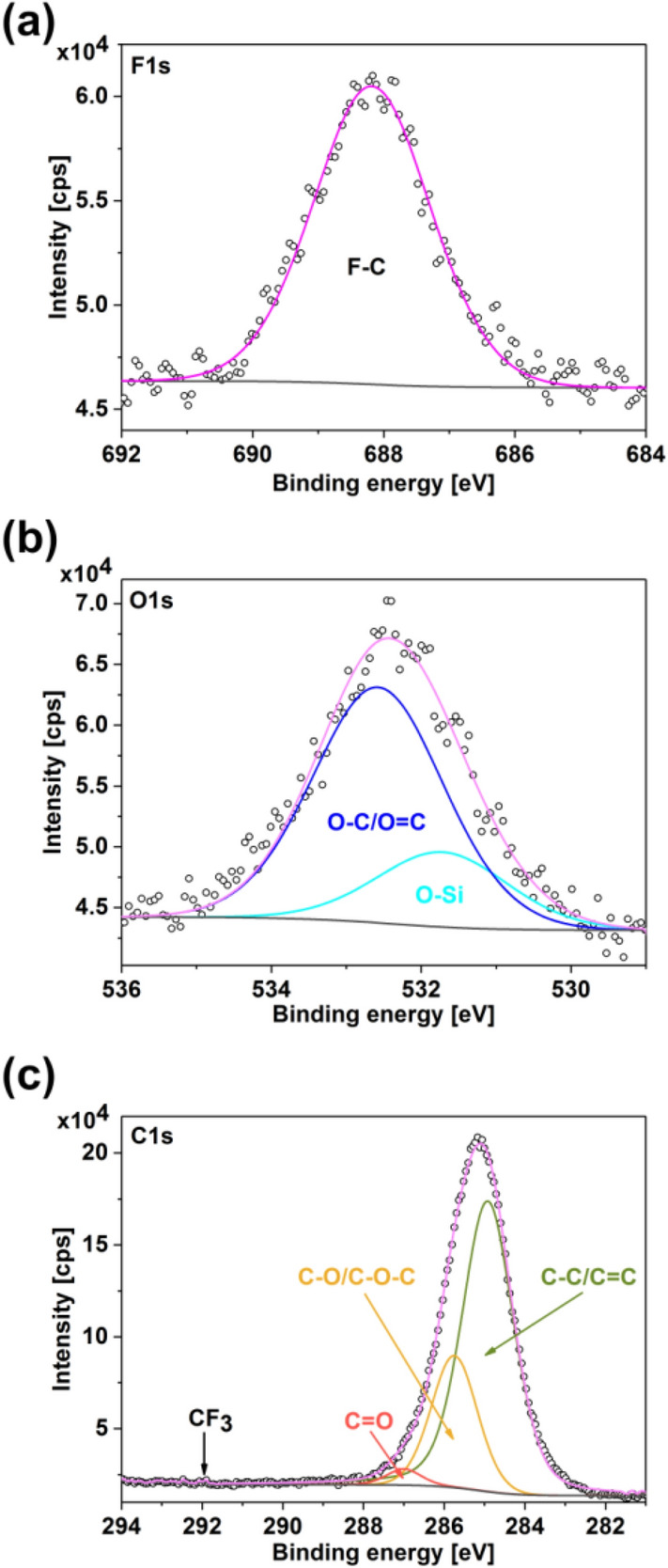
XPS spectrum of the SWCNT dispersion reacted with bis[4-(trifluoromethyl)benzoyl] peroxide recorded in the (**a**) F1s, (**b**) O1s, and (**c**) C1s areas. The O-Si peak comes from the substrate used for the deposition of the modified SWCNT material for analysis.

For complete verification of our hypothesis, we executed additional control experiments. First, we checked the stability of the product after chemical transformation. The saponification reaction proceeds irreversibly, so we measured PL from the sample after 24 h. The same outcome was reached (Fig. 8c), which supported the theory. We also validated whether the expected organic group was attached to the SWCNT sidewall to rule out the possibility of implantation of some sort of an inorganic oxygen functionality by BPO (a well-known oxidizing agent). For this, we employed an analogous BPO reactant labeled by the trifluoromethyl group. The SWCNT dispersion in toluene was reacted with bis[4-(trifluoromethyl)benzoyl] peroxide (CF_3_-BPO), which, if the hypothesis was correct, should produce ester modified SWCNTs (labeled with fluorine) along with certain amounts of trifluoromethylbenzene as a by-product. The side-product was preferentially removed by prolonged heating as its boiling point was lower than that of toluene used as a solvent. Once it was evaporated, we were confident that it would not interfere with the chemical analysis. The modified SWCNTs were investigated by XPS afterward (Fig. 9).

A strong signal of fluorine in the range expected for organic fluorine was detected (Fig. 9a); therefore, we concluded that the desired functional group was successfully implanted. The low intensity of the CF_3_ component expected in the C1s region could be rationalized by substantial C–F bond degradation by the impact of X-radiation^39^. To prove the presence of fluorine in the final material, the F1s signal was recorded before any other part and survey spectra. Furthermore, signals coming from oxygen atoms both in O1s (Fig. 9b) and C1s (Fig. 9c) locations of relatively high intensity could be justified by the way how the excess reactant was removed. A septum was removed after 1 h of reaction time to evaporate this compound. The reaction mixture was then heated for 3 h in an open-system configuration under the influence of atmospheric oxygen. Because the solubility of oxygen in toluene increased with the rise in temperature^40^, we expected that in such configuration, the sample would become over-functionalized with oxygen groups. Nevertheless, the point of this experiment was to prove the presence of fluorine in the final product, which would indicate successful grafting of the surface, so this effect was disregarded. Based on the results presented above, we concluded that unambiguously the SWCNTs were modified by ester groups, which enhanced their light emission capabilities.

## Conclusions

In summary, this contribution gave definite proof that the chemistry of organic radicals could be adapted to generate a new type of traps for mobile excitons in SWCNTs. The thermal dissociation of BPO gave benzoyloxy radicals, which then reacted with SWCNT to produce ester-modified SWCNTs. A range of methods validated the successful modification of the material this way. Firstly, it was found that the position of PL peaks from the functionalized material did not correspond with any of the results in the literature (oxidized, arylated or alkylated SWCNTs). Secondly, the grafted functional group was susceptible to hydrolysis upon exposure to the base as expected for ester functionality. Thirdly, fluorine-labeled BPO analog enabled us to detect fluorine on the SWCNT surface, a direct proof of successful modification by an organic group. In light of these results, we concluded that a benzoate derivative of SWCNTs was created.

The presented methodology combines the benefits of the established techniques of defect implantation in SWCNTs, while simultaneously circumvents their weak points. The transformation is simple to conduct and does not require dangerous chemicals as compared with typical reductive alkylations. Furthermore, in contrast to diazonium chemistry commonly employed for this purpose, it does not take days and does not involve light-sensitive chemicals. Instead, the process engages an oxidizing agent routinely used in organic chemistry, which successfully modifies the optical properties of SWCNTs in just an hour. Lastly, although it similarly quick as recently reported alternative techniques of oxidation, it also offers versatility because a spectrum of organic chemical groups can be attached to the surface via C–O interconnect.

The key benefit of the proposed concept is that various polymers have been shown to have selectivity for the isolation of near-monochiral fractions of SWCNTs in organic solvents. By engaging the routine described herein, one can directly enhance the light emission capabilities of sorted SWCNTs without the need to carry out a surfactant exchange process to make the material water compatible. This approach, therefore, should pave a way towards the creation and application of a multitude of differently modified monochiral SWCNTs in photonics.

## Experimental

### Materials

Single-walled carbon nanotubes (SWCNTs) (CoMoCAT (6,5)-enriched), 2,2′-Azobis(2-methylpropionitrile) (AIBN), benzoyl peroxide (BPO), *meta*-chloroperoxybenzoic acid (mCPBA) (≤ 77%), and *N*-iodosuccinimide (NIS) tetraethylammonium hydroxide solution ~ 25% in methanol (~ 1.5 M) were purchased from Sigma-Aldrich. Bis[4-(trifluoromethyl)benzoyl] peroxide (CF_3_-BPO) was purchased from American Custom Chemicals Corporation. Toluene was purchased from Wako Pure Chemical Industries, Ltd. Poly(9,9-dioctylfluorenyl-2,7-diyl and bipyridine copolymer (PFO-Bpy) was obtained from American Dye Source, Inc. All chemicals were of analytical purity and used without further purification.

### Preparation of SWCNT dispersion in toluene

A bath-type sonicator (BRANSON, CPX5800H-J), a tip-type sonicator (Tomy Seiko, UD-200), and an ultracentrifuge (Hitachi, Himac CS 100 GXL) were used for the preparation of the SWNT dispersion. In a 50 mL glass bottle, 3 mg of the SWCNT material was dispersed in a toluene solution of PFO-Bpy (0.4 mg/mL, 15 mL) and sonicated using a bath-type sonicator for 3 h and then using a tip-type sonicator for 2 h. The resulting dispersion was ultracentrifuged at 10,000 rpm for 1 h. The top 90% of the supernatant was collected for experiments.

### Preparation of modified SWCNTs using radical organic reagents

#### Preliminary studies to select the reactant for the study

The reactions were carried out by combining the SWCNT dispersion obtained above and organic radical reagent solution (AIBN, BPO, *m*CPBA, or NIS) to establish 80 µg/mL concentration of these radical-reservoirs in SWCNT toluene dispersion. Then, the reaction was allowed to proceed for 60 min at 100 °C under septum-sealed conditions. The products of the transformation were characterized by PL spectroscopy immediately after the reaction completion.

#### Kinetic studies using BPO

Time-dependence experiments were performed for 100 µg/mL concentration of BPO. The reactions were carried out by mixing the SWCNTs dispersion and an appropriate amount of BPO stock solution to reach 80 µg/mL in SWCNT toluene dispersion. The reaction was conducted for the specified time at 100 °C under septum-sealed conditions. The reaction course was monitored via PL spectroscopy at different time intervals (15, 30, 45, and 60 min).

#### Concentration screening using BPO

The adjustment of the amount of BPO was necessary to obtain the optimum concentration of defects. We tested BPO concentrations from 1 µg/mL to 1200 µg/mL in SWCNT toluene dispersion at 100 °C. The reactions were carried out in the same way as described above (100 °C, 60 min process time). The reactions were monitored via PL spectroscopy after 60 min.

#### Basic hydrolysis of modified SWCNTs

The obtained dispersions of modified-SWCNTs after exposure to 500 µg/mL of BPO (1 mL) were treated with 2 µL of a methanol solution of tetraethylammonium hydroxide (1.5 M). The reactions were carried out by mixing the components at room temperature for the specified time. 2D PLE monitored the experiments in selected time intervals (15 min, and 24 h).

#### *Control reaction with CF*_*3*_*-BPO*

SWCNT dispersion was exposed to CF_3_-BPO adjusted to reach the concentration of 100 µg/mL at 100 °C for 1 h under septum-sealed conditions, as previously described. After this time, the septum was removed, and 2/3 of the reaction volume was evaporated by heating the mixture for another 3 h. Trifluoromethylbenzene has a boiling point of 101–103 °C^41^, which is lower than that of toluene 111 °C^41^, so it can be preferentially removed by evaporation. After the concentration step, the dispersion was deposited onto a p-type doped silicon substrate for XPS analysis.

### Characterization

#### Optical properties of modified SWCNTs

Visible-near-infrared absorption (Vis/NIR, 400–1600 nm) and photoluminescence (PLE, excitation 500–800 nm, emission 815–1600 nm) spectra were measured using a V-670 (JASCO) and a HORIBA JOBIN YVON spectrofluorometer (FluorologR-3 with FluorEssence), respectively. Quartz cells with a 1-cm-path length were used for the optical measurements. The analysis was performed at room temperature. 2D PLE maps were normalized to the maxima of the peaks with the highest intensity. Raman spectra were measured using a Raman spectrometer (RAMANtouch, Nanophoton Corporation) at an excitation wavelength of 633 nm at room temperature.

#### Chemical composition of modified SWCNTs

X-ray photoemission spectra (XPS) were acquired using PREVAC EA15 hemispherical electron energy analyzer coupled with an X-ray source (PREVAC dual-anode XR40B, Al-Ka line, energy 1486.60 eV) as well as a 2D-MCP detector. The measurements were conducted at the following conditions: 0.9 eV scanning step, 200 eV pass energy, and under 2 × 10^−8^ Pa system base pressure. The binding energy (BE) scale was calibrated by positioning the reference peaks of Au 4f_7/2_ (84.0 eV). The obtained data were fitted using CASA XPS embedded algorithms as well as relative sensitivity factors. The background was modeled by the Shirley function and subtracted. The estimated uncertainty for the energy position of components is on the level of 0.1 eV.

## Supplementary information

Supplementary Figures.
