# Supplementary material for: Enhancing near-infrared photoluminescence from single-walled carbon nanotubes by defect-engineering using benzoyl peroxide
Source: Sci Rep. 2020 Nov 16;10:19877. doi: 10.1038/s41598-020-76716-9 (PMC7669876; doi:10.1038/s41598-020-76716-9)
Supplement: Supplementary file 1 — Supplementary Figures. [file 41598_2020_76716_MOESM1_ESM.pdf]

Supplementary Information

# Enhancing near-infrared photoluminescence from single-walled carbon nanotubes by defect-engineering using benzoyl peroxide

Lukasz Przypis[a], Maciej Krzywiecki[b], Yoshiaki Niidome[c], Haruka Aoki[c], Tomohiro Shiraki[c], Dawid Janas[a,*]

[a] Department of Organic Chemistry, Bioorganic Chemistry and Biotechnology, Silesian University of Technology, B. Krzywoustego 4, 44-100 Gliwice, Poland

[b] Institute of Physics-CSE, Silesian University of Technology, Konarskiego 22B, 44-100 Gliwice, Poland

[c] Department of Applied Chemistry, Graduate School of Engineering, Kyushu University, 744 Motooka, Nishi-ku, 819-0395 Fukuoka, Japan

*Corresponding author: Dawid.Janas@polsl.pl

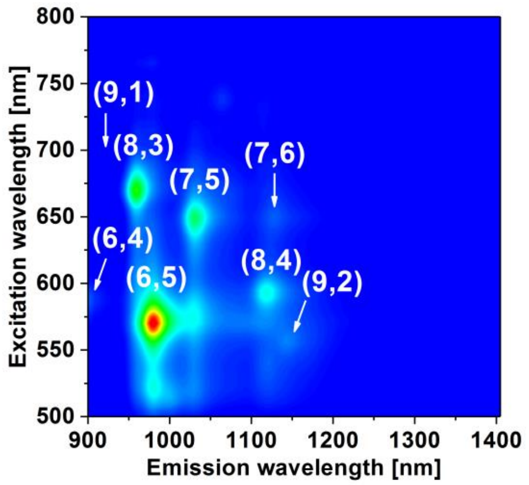


**Figure S1** 2D PLE map of (6,5)-enriched SWCNTs dispersed in 2% sodium cholate aqueous solution.

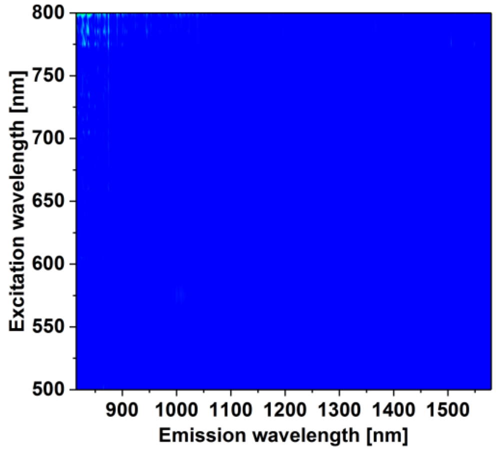


**Figure S2** 2D PLE map of (6,5)-enriched SWCNTs dispersed in 2% sodium cholate aqueous solution after treatment with BPO at 100°C for 1h in water.

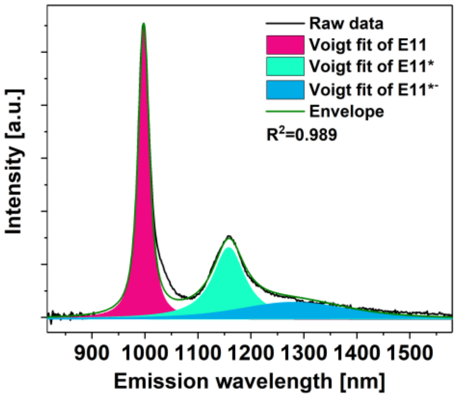


**Figure S3** Deconvolution of an exemplary PL spectrum of (6,5) SWCNT dispersion in PFO-Bpy/toluene solution after the treatment with BPO at 60 µg/mL at 100°C for 1h. Voigt profile was utilized for the resolution of the $E_{11}$, $E_{11}^{*}$, and $E_{11}^{*-}$ signatures. The integrated area under these curves was used for analysis in Figs. 5b, 6b-c.

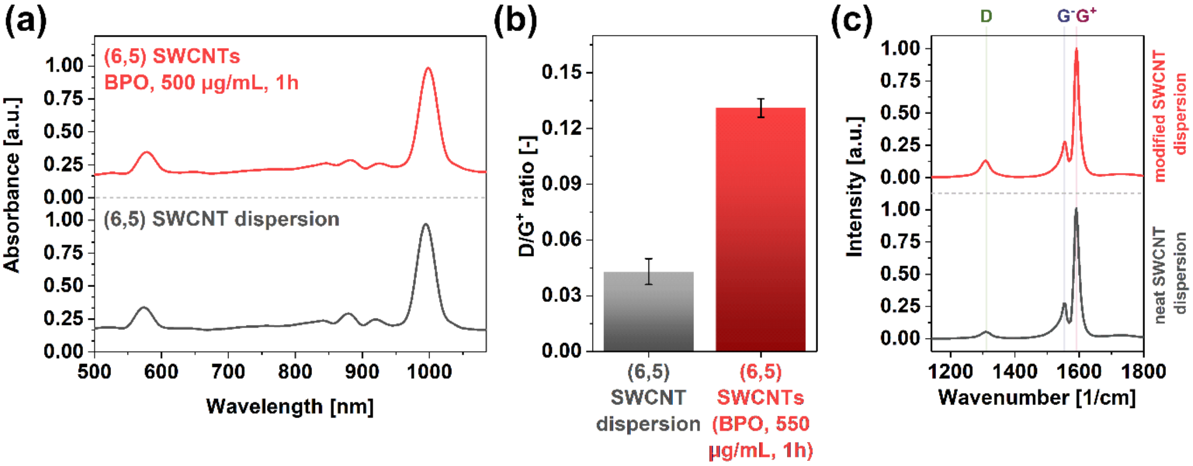


**Figure S4** (a) Absorption spectra of (6,5) SWCNT dispersion in PFO-Bpy/toluene solution before (grey line) and after the treatment with BPO at 500 µg/mL at 100°C for 1h (red line). (b) D/G+ ratios measured by Raman spectroscopy and (c) corresponding Raman spectra.

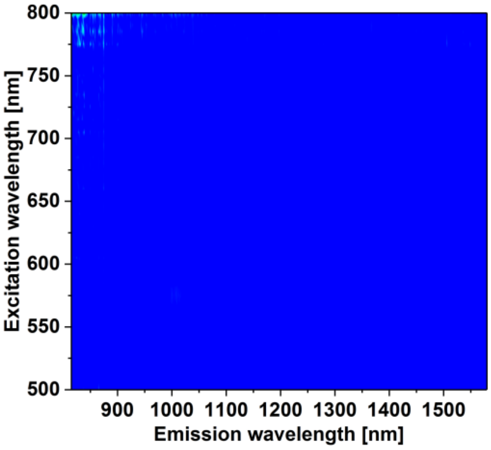


**Figure S5** 2D PLE map of BPO in PFO-Bpy and toluene mixture in the absence of SWCNTs.
